# Comorbidity profiles of chronic migraine sufferers in a national database in Taiwan

**DOI:** 10.1007/s10194-012-0447-4

**Published:** 2012-04-12

**Authors:** Yong-Chen Chen, Chao-Hsiun Tang, Kwong Ng, Shuu-Jiun Wang

**Affiliations:** 1 National Defense Medical Center, Graduate Institute of Life Sciences, Taipei, Taiwan; 2 School of Health Care Administration, Taipei Medical University, Taipei, Taiwan; 3 Regional Health Outcomes, Allergan Singapore Pte Ltd, Singapore, Singapore; 4 Taipei Veterans General Hospital, Neurological Institute, Taipei, Taiwan; 5 National Yang-Ming University School of Medicine, Taipei, Taiwan; 6 Department of Neurology, Taipei Veterans General Hospital, Neurological Institute, #201, Shi-Pai Road, Section 2, Taipei, 112 Taiwan

**Keywords:** Chronic migraine, Comorbidity, Taiwan

## Abstract

Chronic migraine (CM; ≥15 headache days per month, ≥3 months) is associated with a higher prevalence of comorbidities than episodic migraine (<15 headache days per month). However, it is unclear whether a similar pattern exists in Asian patients. To examine this, a retrospective matched cohort study was conducted using the Taiwan National Health Insurance Research Database. CM cases were defined as patients with at least one neurological outpatient visit with a primary or secondary ICD-9-CM (International Classification of Diseases, Ninth Revision, Clinical Modification) code of 346.11, diagnosed by neurologists at medical centers during 2007–2008. The study group was compared with patients suffering from other migraine subtypes and non-migraineurs in the general population. Both comparison groups were matched with CM sufferers at a 4:1 ratio by age, gender, urbanization level of the residence, income, and hospital setting. Relative risk (RR) was calculated using conditional logistic regression. Compared with patients with other migraines (*n* = 2,226), CM sufferers (*n* = 681) had a higher risk of hyperlipidemia (RR = 1.32; *P* = 0.041), asthma (RR = 1.77; *P* = 0.007), depression (RR = 1.88; *P* < 0.0001), bipolar disorder (RR = 1.81; *P* = 0.022) and anxiety disorders (RR = 1.48; *P* = <0.0001). Compared with the non-migraineurs (*n* = 3,790), CM sufferers (*n* = 948) had significantly increased risks of cardiovascular disease, sinusitis, asthma, gastrointestinal ulcers, vertigo and psychiatric disorders by 1.6–3.9-fold. In conclusion, CM is associated with significant comorbidities in Asian patients. Differences in the comorbidity profiles of CM compared with other migraines have highlighted that patients with CM differ not just in terms of headache frequency but also in other important aspects.

## Introduction

Migraine is a common neurological disorder affecting 9.1 % of the general population in Taiwan [1]. It can be classified into two subgroups based on headache frequency, i.e. episodic migraine (EM; <15 headache days per month) and chronic migraine (CM, ≥15 headache days per months, ≥3 months) [2, 3]. In the second edition of the International Classification of Headache Disorders (ICHD-2), CM is listed as a complication of EM and is defined as a diagnosis of migraine with 15 or more headache days per month over the past 3 months, of which at least eight headache days meet the criteria for migraine without aura or respond to migraine-specific treatment [2, 3].

It is estimated that EM sufferers develop CM at the rate of 2.5 % per year [4]. The prevalence rate of CM in Taiwan is estimated to be 1.7 %, with a female to male ratio of approximately 2:1 [5]. Previous studies have demonstrated that, when compared to EM, CM was associated with greater disability, poorer productivity, lower quality of life and higher healthcare expenditures [6–9].

Migraine has been reported to be associated with various comorbidities. Previously examined comorbidities include metabolic syndrome (obesity, stroke, subclinical vascular brain lesions, coronary artery disease and hypertension), psychiatric disorders (depression, anxiety, bipolar disorder, panic disorder and suicide), epilepsy, asthma and other disorders [10]. Recently, the American Migraine Prevalence and Prevention (AMPP) study, a large, longitudinal, population-based survey involving 24,000 patients with headaches, demonstrated that patients with CM differed from the EM population in several aspects in terms of the sociodemographic and comorbidity profiles [11]. However, it is unclear if a similar pattern exists in Asian patients. The present study therefore aims to provide insight into the prevalence of comorbidities in patients with CM in Taiwan.

## Methods

### Study design and data source

A population-based retrospective matched cohort study was conducted utilizing the National Health Insurance Research Database (NHIRD) in Taiwan. The National Health Insurance (NHI) is a social health insurance program that covers nearly 99 % of the population for comprehensive health care in Taiwan. The NHIRD contains all benefit claims and was established by the Taiwan National Health Research Institute (NHRI), in cooperation with the Bureau of NHI, for research purposes.

In this study, patients with migraine were sampled from the NHIRD inpatient and outpatient expenditure claims data files from the year 2006 to 2009. These files contained the birth date and gender of the patient, services provided, principal and secondary diagnosis codes, and itemized expenditure on the various types of services, such as physician consultation, drugs and prescription services, and in-hospital care. One million NHI beneficiaries, representative of the non-migraineurs in Taiwan, were taken from a similar database, from March 1995 when the NHI was put into effect, until the end of 2009.

All personal identifiers were encrypted by the NHRI before they were released to the researchers. Thus confidentiality was assured according to the data regulations of the Bureau of NHI, and the approval of the Institutional Review Board was not required.

### The study cohort and the comparison cohorts

Patients with migraine were either assigned as patients with CM or patients suffering from migraine other than CM. The study cohort consisted of CM cases, defined as patients with at least one neurological outpatient visit with primary or secondary 2007 ICD-9-CM (International Classification of Diseases, Ninth Revision, Clinical Modification) code of 346.11 (common migraine with intractable migraine so stated), and who were diagnosed by a certified neurologist at a medical centre between 2007 and 2008 (*n* = 1,101). As there was no known ICD-9-CM code for CM diagnosis at that time, these criteria were used to capture CM cases more accurately, based on our knowledge of where the patients were treated.

Patients suffering from migraine other than CM were defined as patients who had at least one outpatient visit with primary or secondary ICD-9-CM codes for migraine 346.XX other than 346.11 between 2007 and 2008, and who had never been diagnosed with migraine prior to 1 January 2007. These included 2007 ICD diagnostic codes for classical migraine without intractable migraine (346.00), common migraine without intractable migraine (346.10), variants of migraine without intractable migraine (346.20), other forms of migraine without intractable migraine (346.80) and migraine unspecified without intractable migraine (346.90). Any cases with a diagnostic code involving intractable migraine were excluded. This group of patients formed our first comparison cohort and was matched with the study cohort at a 4:1 ratio by age, gender and hospital setting. The index date was defined as the date when the study cases were first diagnosed as CM and when patients with migraine other than CM were diagnosed.

The second comparison cohort was drawn from the 2005 to 2009 database of 1,000,000 NHI beneficiaries who were never diagnosed with migraine. They were matched with the study sample at a 4:1 ratio by age, gender, urbanization level of the residence, and income (*n* = 3,790). The date when the study cases were diagnosed with CM was assigned as the index date for both groups.

A total of 420 CM cases that were not matched with other migraine sufferers and 153 CM cases that were not matched with non-migraineurs were lost from the study. The final sample sizes obtained for the matched cohorts were 681 and 948, respectively.

### Definition of urbanization level of the residence

According to Taiwan National Health Research Institute publications, urbanization levels in Taiwan are divided into seven strata, with level 1 referring to the “most urbanized” and level 7 referring to the “least urbanized” communities. As there were very small numbers of CM cases in levels 4, 5, 6 and 7, these four levels were combined into level 4–5 and level 6–7 groups [12].

### Definition of comorbidity

Patients who made at least three claims for outpatient visits or one claim for hospitalization within 1 year after the index date with principal/secondary diagnoses of the following diseases were considered probable comorbidities associated with CM. These diseases can generally be categorized as:

1. metabolic syndrome, including hypertension (ICD-9-CM codes 401.0, 401.1, 401.9, 402–405, 437.2), diabetes (ICD-9-CM code 250), hyperlipidemia (ICD-9-CM codes 272.0–272.4), heart disease (ICD-9-CM codes 410–429), and ischemic strokes (ICD-9-CM codes 433, 434 and 436)
2. respiratory diseases, including sinusitis (ICD-9-CM codes 461, 473), asthma (ICD-9-CM code 493), and emphysema or chronic obstructive pulmonary disease (COPD) (ICD-9-CM codes 490–492, 496)
3. gastrointestinal disorders, including peptic ulcer (ICD-9-CM codes 531–534), stomach or duodenal ulcers (ICD-9-CM codes 536–537) and gastrointestinal hemorrhage (ICD-9-CM codes 578)
4. neurological disorders, including epilepsy (ICD-9-CM code 345) and vertigo (ICD-9-CM codes 780.4, 386)
5. psychiatric disorders, including drug abuse (ICD-9-CM codes 292, 304, 305.1–305.9), depression (ICD-9-CM codes 296.2, 296.3, 296.9, 300.4, 309.0, 309.1, 311), bipolar disorder (ICD-9-CM codes 296.0, 296.1, 296.4–296.7, 296.80, 296.89), anxiety disorders (ICD-9-CM codes 300.0–300.3, 300.5–300.9, 309.2, 309.4, 309.81, 313.0), panic disorder (ICD-9-CM codes 300.01, 300.21, 300.22), obsessive compulsive disorder (ICD-9-CM code 300.3), suicide (ICD-9-CM codes E950–E959) and insomnia (ICD-9-CM codes 307.41, 307.42, 307.49, 780.50, 780.52, 780.55, 780.56, 780.59).

### Statistical analysis

Chi-square tests were performed to analyze the sociodemographic characteristics of the sample groups. Prevalence of comorbidities within 1 year after the index date and their RRs with 95 % confidence interval (CI) were calculated using conditional logistic regression analysis. The RRs were adjusted for age, gender, urbanization level of the residence and income. Each comorbid disease was compared independently. A *P* value of <0.05 was considered to be statistically significant. All analyses were performed using the SAS system for Windows, version 9.2 (SAS Institute, Cary, NC).

## Results

Table 1 shows the demographic characteristics of the study group and the two comparison cohorts. All demographic characteristics were well matched in the two comparison cohorts. The majority of CM cases were female (approximately 80 %) and were mostly in the young adults and middle-aged groups, ranging from age 20 to 59 years.

**Table 1:** Sociodemographic characteristics of patients with chronic migraine (CM) and the two comparison groups

	CM cases (*N* = 681)		Comparison cohort: cases with other migraine (*N* = 2,226)		*P* value	CM cases (*N* = 948)		Comparison cohort: non-migraine population (*N* = 3,790)		*P* value
	*n*	%	*n*	%		*n*	%	*n*	%	
Age (years)										
<20	40	5.87	102	4.58	0.348	70	7.38	280	7.39	1.000
20–40	273	40.09	941	42.27		382	40.30	1,526	40.26	
40–60	292	42.88	964	43.31		356	37.55	1,424	37.57	
≥60	76	11.16	219	9.84		140	14.77	560	14.78	
Gender										
Male	146	21.44	446	20.04	0.426	234	24.68	934	24.64	0.980
Female	535	78.56	1,780	79.96		714	75.32	2,856	75.36	
Urbanization level										
1 (high)	259	38.03	934	41.96	0.101	282	29.75	1,128	29.76	1.000
2	229	33.63	768	34.50		305	32.17	1,220	32.19	
3	82	12.04	252	11.32		151	15.93	604	15.94	
4–5	70	10.28	187	8.40		109	11.50	436	11.50	
6–7	35	5.14	79	3.55		100	10.55	400	10.55	
Missing	6	0.88	6	0.27		1	0.11	2	0.05	
Premiums (NT$)										
>43,900	79	11.60	242	10.87	0.722	103	10.86	412	10.87	1.000
30,301–43,900	55	8.08	187	8.40		81	8.54	324	8.55	
21,001–30,300	74	10.87	274	12.31		105	11.08	420	11.08	
≤21,000	67	9.84	241	10.83		98	10.34	392	10.34	
Fixed	406	59.62	1,282	57.59		561	59.18	2,242	59.16	

*RR* relative risk, *CI* confidence interval, *SD* standard deviation

Table 2 presents the distribution and RR of comorbidities among patients with CM and the two comparison cohorts. Compared with other migraines, CM was associated with a significantly higher risk of hyperlipidemia (RR = 1.32; 95 % CI = 1.01–1.72), asthma (RR = 1.77, 95 % CI = 1.17–2.69), depression (RR = 1.88, 95 % CI = 1.51–2.33), bipolar disorder (RR = 1.81, 95 % CI = 1.09–2.99) and anxiety disorders (RR = 1.48, 95 % CI = 1.19–1.83). No significant associations were seen between CM and other cardiovascular, respiratory, gastrointestinal, neurological or psychiatric comorbidities compared to other migraines.

**Table 2:** Association of comorbidities in patients with chronic migraine (CM) versus other migraine and non-migraine population

Comorbidity	No. of comorbidities				Cases with CM versus other migraine			No. of comorbidities				Cases with CM versus non-migraine population			Cases with other migraine versus non-migraine population		
	Cases with CM		Cases with other migraine		Adjusted RR	95 % CI	*P* value	Cases with CM		Non-migraineurs		Adjusted RR	95 % CI	*P* value	Adjusted RR	95 % CI	*P* value
	*n*	%	*n*	%				*n*	%	*n*	%						
	681		2,226					948		3,790							
Metabolic syndrome																	
Hypertension	115	16.89	294	13.21	1.24	(0.99–1.55)	0.064	173	18.25	462	12.19	1.64	(1.35–1.99)	<0.0001	1.17	(1.02–1.35)	0.022
Diabetes	32	4.7	95	4.27	1.08	(0.75–1.56)	0.684	42	4.43	199	5.25	0.85	(0.62–1.18)	0.334	0.98	(0.79–1.22)	0.863
Hyperlipidemia	67	9.84	160	7.19	1.32	(1.01–1.72)	0.041	88	9.28	177	4.67	1.84	(1.45–2.32)	<0.0001	1.30	(1.10–1.53)	0.002
Heart disease	57	8.37	148	6.65	1.18	(0.89–1.58)	0.252	84	8.86	185	4.88	1.73	(1.36–2.21)	<0.0001	1.32	(1.11–1.58)	0.002
Ischemic strokes	14	2.06	63	2.83	0.74	(0.43–1.28)	0.284	24	2.53	46	1.21	1.81	(1.18–2.78)	0.006	1.85	(1.41–2.42)	<0.0001
Respiratory diseases																	
Sinusitis	60	8.81	156	7.01	1.17	(0.89–1.53)	0.253	90	9.49	182	4.8	1.77	(1.41–2.21)	<0.0001	1.23	(1.05–1.46)	0.013
Asthma	24	3.52	33	1.48	1.77	(1.17–2.69)	0.007	33	3.48	44	1.16	2.27	(1.59–3.25)	<0.0001	1.30	(0.92–1.83)	0.145
Emphysema or COPD	19	2.79	41	1.84	1.40	(0.87–2.26)	0.165	28	2.95	58	1.53	1.73	(1.16–2.58)	0.007	1.31	(0.95–1.80)	0.099
Gastrointestinal disorders																	
Peptic ulcer	79	11.6	204	9.16	1.22	(0.95–1.56)	0.113	109	11.5	152	4.01	2.39	(1.93–2.95)	<0.0001	1.59	(1.37–1.85)	<0.0001
Stomach and duodenal ulcer	53	7.78	131	5.88	1.18	(0.89–1.57)	0.262	86	9.07	117	3.09	2.31	(1.84–2.91)	<0.0001	1.43	(1.19–1.71)	<0.0001
Gastrointestinal hemorrhage	4	0.59	5	0.22	1.94	(0.69–5.46)	0.209	4	0.42	11	0.29	1.35	(0.50–3.68)	0.557	0.96	(0.39–2.33)	0.927
Neurological disorders																	
Epilepsy	6	0.88	31	1.39	0.60	(0.27–1.35)	0.218	10	1.05	18	0.47	1.82	(0.97–3.44)	0.063	1.75	(1.21–2.51)	0.003
Vertigo	29	4.26	99	4.45	0.89	(0.61–1.30)	0.549	43	4.54	50	1.32	2.49	(1.81–3.43)	<0.0001	1.82	(1.47–2.24)	<0.0001
Psychiatric disorders																	
Drug abuse	1	0.15	1	0.04	2.30	(0.32–16.4)	0.406	0	0	0	0	–	–	–	1.66	(0.24–11.73)	0.612
Depression	104	15.27	157	7.05	1.88	(1.51–2.33)	<0.0001	120	12.66	64	1.69	3.83	(3.14–4.68)	<0.0001	1.88	(1.59–2.22)	<0.0001
Bipolar disorder	16	2.35	22	0.99	1.81	(1.09–2.99)	0.022	18	1.9	7	0.18	3.88	(2.39–6.29)	<0.0001	1.90	(1.23–2.93)	0.004
Anxiety disorders	106	15.57	220	9.88	1.48	(1.19–1.83)	<0.0001	122	12.87	122	3.22	2.89	(2.37–3.52)	<0.0001	1.74	(1.51–2.01)	<0.0001
Panic disorder	8	1.17	13	0.58	1.54	(0.76–3.13)	0.233	13	1.37	11	0.29	2.85	(1.62–5.00)	<0.0001	1.23	(0.71–2.12)	0.465
Obsessive compulsive disorder	1	0.15	4	0.18	0.94	(0.13–6.80)	0.952	1	0.11	3	0.08	1.25	(0.17–9.02)	0.822	2.16	(0.80–5.83)	0.128
Suicide	0	0	0	0	–	–	–	0	0	0	0	–	–	–		–	
Insomnia	96	14.1	250	11.23	1.21	(0.97–1.51)	0.094	122	12.87	179	4.72	2.28	(1.87–2.78)	<0.0001	1.58	(1.38–1.81)	<0.0001

*RR* relative risk, *CI* confidence interval, *COPD* chronic obstructive pulmonary disease

Compared with non-migraineurs, CM sufferers had a 1.6–3.9-fold risk of comorbidities, including hypertension (RR = 1.64, 95 % CI = 1.35–1.99), hyperlipidemia (RR = 1.84, 95 % CI = 1.45–2.32), heart disease (RR = 1.73, 95 % CI = 1.36–2.21), ischemic stroke (RR = 1.81, 95 % CI = 1.18–2.78), sinusitis (RR = 1.77, 95 % CI = 1.41–2.21), asthma (RR = 2.27, 95 % CI = 1.59–3.25), emphysema or COPD (RR = 1.73, 95 % CI = 1.16–2.58), peptic ulcers (RR = 2.39, 95 % CI = 1.93–2.95), duodenal ulcers (RR = 2.31, 95 % CI = 1.84–2.91), vertigo (RR = 2.49, 95 % CI = 1.81–3.43), and psychiatric disorders, including depression (RR = 3.83, 95 % CI = 3.14–4.68), bipolar disorder (RR = 3.88, 95 % CI = 2.39–6.29), anxiety disorders (RR = 2.89, 95 % CI = 2.37–3.52), panic disorder (RR = 2.85, 95 % CI = 1.62–5.00), and insomnia (RR = 2.28, 95 % CI = 1.87–2.78). Significant associations were not found between CM and diabetes, gastrointestinal hemorrhage, epilepsy and obsessive compulsive disorder.

Additionally, we also compared the RR of comorbidities between the two comparison cohorts. Compared with non-migraineurs, the other migraine sufferers had a 1.2–1.9-fold risk of comorbidity. In general, the RRs were numerically lower for the other migraine versus non-migraineurs compared with the CM sufferers versus non-migraineurs, except for epilepsy and ischemic stroke. Other migraine was associated with a significantly higher risk of epilepsy (RR = 1.75, 95 % CI = 1.21–2.51) and ischemic stroke (RR = 1.85, 95 % CI = 1.41–2.42).

## Discussion

The present study found that patients with CM had a significantly higher risk of hyperlipidemia (RR = 1.32), asthma (RR = 1.77), and anxiety disorders, depression and bipolar disorder (RR = 1.48–1.88) than those with other migraines. However, the prevalence of gastrointestinal, neurological and metabolic (other than hyperlipidemia) comorbidities in patients with CM and patients with other migraines were similar. The study also confirmed that CM posed a higher risk for hypertension (RR = 1.64), hyperlipidemia (RR = 1.84), heart disease (RR = 1.73) and ischemic stroke (RR = 1.81) compared with non-migraineurs. The RR was also significantly higher for sinusitis (RR = 1.77), asthma (RR = 2.27), and emphysema or COPD (RR = 1.73), and CM sufferers were also found to be more likely to develop peptic ulcer (RR = 2.39), stomach/duodenal ulcer (RR = 2.31) and vertigo (RR = 2.49). The RR for psychiatric disorders in patients with CM was 3.83 for depression, 3.88 for bipolar disorder, 2.89 for anxiety disorders, 2.85 for panic disorder and 2.28 for insomnia.

### Metabolic syndrome

Metabolic syndrome is a constellation of risk factors that contribute to the development of cardiovascular disease, cerebrovascular disease and diabetes mellitus. Scher et al. [13] reported that migraineurs had an increased risk of hypertension (odds ratio, OR = 1.63, 95 % CI = 1.2–2.1), elevated total cholesterol (>240 mg/dL) (OR = 1.22, 95 % CI = 1.0–1.5), and were more likely to have a parental history of early myocardial infarction (OR = 2.25, 95 % CI = 1.0–5.1). Kurth et al. [14] also found that women with any history of migraine had an increased risk of myocardial infarction (hazard ratio, HR = 1.41, 95 % CI = 1.03–1.91) and coronary revascularization (HR = 1.35, 95 % CI = 1.09–1.67). Results from a meta-analysis by Schürks et al. [15] showed that patients with migraine were almost twice as likely to have ischemic stroke (pooled RR = 1.73, 95 % CI = 1.31–2.29), especially patients suffering from migraine with aura. We also observed a higher prevalence of hypertension, hyperlipidemia, heart disease and ischemic stroke in patients with CM compared to the non-migraineurs. As the methodology used in our study only allowed assessment of comorbidities within the first year of the index date, a longer duration of follow-up would be necessary to assess the correlation of CM and metabolic syndrome.

### Respiratory diseases

Findings from a Norwegian study indicated that migrainous headaches were related to asthma, hay fever and chronic bronchitis and this association increased with headache frequency, particularly in migraines occurring 15 days or more per month [16]. Similarly, the AMPP study showed that asthma (OR = 1.53, 95 % CI = 1.27–1.84) was more likely to be reported by those with CM compared with episodic migraineurs [11]. Our study confirmed that CM displayed a higher risk of asthma compared with patients with other migraine (RR = 1.77). The prevalence of sinusitis (RR = 1.77), asthma (RR = 2.27) and COPD, including emphysema (RR = 1.73) were significantly higher than seen in non-migraineurs. Davey et al. [17] reported a similar RR of asthma in patients with migraine (RR = 1.59, 95 % CI = 1.54–1.65), and COPD (RR = 1.22, 95 % CI = 1.12–1.34), although the diagnostic codes used for migraine in the Davey study differed from those used in our present study. We found no significant association of CM with other respiratory disorders, such as sinusitis or COPD (including emphysema), when looking at the CM group compared with the other migraine group; however, this should be confirmed with a longitudinal follow-up in future studies since asthma is a known risk factor for COPD and other pulmonary disorders.

### Gastrointestinal disorders

There are few studies that report an association between migraine and gastrointestinal disorders. Ferrari et al reported that ‘gastrointestinal diseases’ was the second most frequent comorbid disorder in CM sufferers [18]. Results from the present study indicated that patients with CM experienced a higher risk of peptic ulcer (RR = 2.39) and duodenal ulcer (RR = 2.31) compared with the non-migraineurs. Stress is known to provoke the onset of clinically symptomatic migraine, amplifying migraine attack intensity and the duration of the episode [19]. Additionally, stress is often considered a risk factor for upper gastrointestinal tract disease [20]. The high prevalence of CM and peptic ulcer disease comorbidity in our study cohort may be attributed to their shared etiology. Moreover, the increased risk of peptic ulcer and duodenal ulcer may not be caused by migraine itself but by the consequence of increased intake of non-steroidal anti-inflammatory drugs for pain control [8, 21].

### Neurological disorders

The pathophysiological mechanism and clinical features of epilepsy and migraine are postulated to be the same, and one may precipitate, follow or occur concurrently with the other [22]. However, published evidence of the relationship between migraine and epilepsy is controversial, mainly because of the variability of methodology and migraine classification. While some studies have confirmed a comorbid occurrence of migraine among epilepsy patients, others have reported differently [23–26]. Results of our study indicated that there was no significant association between CM and epilepsy.

Our results showed a higher risk of vertigo in patients with CM than in the non-migraineurs (RR = 2.49, 95 % CI = 1.81–3.43). This was consistent with a previous finding by Eggers et al. [27] indicating a strong relationship between vertigo and migraine [27]. Calhoun et al. [28] suggested that vertigo may be considered as an independent entity occurring concurrently in patients with migraine, or as a presenting symptom of migraine itself, particularly in migraineurs with aura. Phillips et al. [29] argued that true vertigo occurring together with migraine is rare; “dizziness” is often mistaken for vertigo, but these entities differ clinically and biologically as vertigo is a hallmark of inner ear disease. Here, we see the importance of differentiating peripheral vestibular vertigo from migrainous vertigo while reviewing available literature on this topic.

### Psychiatric disorders

The relationship between depression and migraine appears to be bidirectional, where one disease entity increases the risk of the development of the other, and vice versa. For example, Breslau et al. [30] found that patients with migraine were predisposed to an increased risk of depression (OR = 5.8) and patients with depression were at higher risk of developing migraine (OR = 3.4), when compared with patients who suffered from other severe headaches or none at all30. Previous studies also showed that migraine was associated with anxiety disorder (OR = 3.9–5.3) [31, 32], panic disorder (OR = 3.7) [33], bipolar disorder (OR = 2.4–7.3) [34] and suicide attempts (OR = 1.6–3.0) [35]. The AMPP study showed that patients with CM were more likely to suffer from anxiety (OR = 1.8), depression (OR = 2.0) and bipolar disorder (OR = 1.56) than those with episodic migraine [11]. Results from the present study confirmed that psychiatric disorder had a greater likelihood to co-exist with CM than with other migraines. A higher risk of psychiatric disorder was evident in patients with CM when compared with the non-migraineurs. Finally, the present study also confirmed findings from previous studies that reported an association between insomnia and migraine [35–37]. The RR for suicide in patients with CM was not obtained in our present study due to lack of data, but a population survey conducted by Wang et al. [38] among adolescents in Taiwan demonstrated that CM with aura was an independent factor for suicide risk, but not migraine without aura. Although our study found no significant link between CM sufferers and illicit drug abuse, analgesia overuse and substance dependence have been noted to be common in patients with CM [39, 40].

The AMPP study by Buse et al. [11] reported that, in addition to lower socioeconomic status and functioning, patients with CM were more likely to have heart disease (OR = 1.4) and cardiac risk factors, such as hypertension, high cholesterol and obesity (OR = 1.2–1.5), stroke (OR = 1.7), ulcers (OR = 1.9) and respiratory diseases, such as allergies, asthma, sinusitis, bronchitis and COPD (OR = 1.4–2.0). Chronic pain disorders and psychiatric disorders such as depression, anxiety and bipolar disorders were more likely to be reported in patients with CM than in those with EM. There was no significant association with diabetes and epilepsy in patients with CM or EM. It should be noted that while Buse et al. looked at the relationship between migraine frequency and comorbidities, our study design looked at the comorbidity profile of patients with CM versus other migraine subtypes and non-migraineurs. Nevertheless, we were able to confirm the findings by Buse et al. that high rates of comorbid diseases were indeed associated with CM, especially hypertension, high cholesterol, heart disease, sinusitis, asthma, COPD, depression, and anxiety and bipolar disorders. Both studies showed no relationship between CM and diabetes, regardless of frequency or subtypes. Our study found that peptic ulcer disease, vertigo, panic disorder and insomnia were significantly evident in patients with CM compared with non-migraineurs. In contrast to the report by Buse et al., our results did not show the association between CM and ischemic stroke.

### CM versus non-migraineurs, and other migraineurs versus non-migraineurs

A comparison of the other migraine patients with non-migraineurs concerning comorbidity would be helpful to decide if the CM sufferers have a different profile as other migraines. In general, the RRs are numerically higher for the former than the latter except for epilepsy and ischemic stroke. However, the reason for this is unknown. Therefore, the data suggest that CM had a higher comorbidity than other migraineurs when compared to controls.

The strengths of this study were mainly attributed to (1) the large population-based sample size to power the study, (2) the comprehensiveness of data within the NHIRD, (3) the use of specific ICD diagnostic codes to define comorbid diseases and (4) the control of confounding variables by matching the study groups according to age, sex and socioeconomic status. The last variable in this study was especially matched by controlling the level of urbanization and personal incomes. Since these factors are associated with medical seeking behaviors, which may in turn influence the chance of disease diagnoses, our comorbidity results are, therefore, more robust for a claim-based dataset. Several limitations should be taken into account when interpreting the results of this study. Firstly, the prevalence of comorbidities was assessed by diagnostic coding in an administrative claims database, and reporting bias may be present due to either over-reporting or under-reporting of the disease. Secondly, there is also a possibility of reporting bias due to our sampling method. Thirdly, because the primary and secondary diagnoses of CM and other medical conditions were not analyzed separately from the database, caution should be exercised when interpreting their association either as distinct concurrent diseases, or as events preceding or following the index disease. Furthermore, the matching was not complete between CM and EM. Finally, there were no corresponding ICD-9-CM codes for CM until 2011. To further define such cases, in this study we used ICD-9 CM code 346.11 (common migraine with intractable migraine so stated) and we only selected cases diagnosed by neurologists at the medical centers. With the new 2011 ICD-9-CM code for CM, our findings may be open to assessment bias due to overlapping selection criteria when defining CM in this study. With the newly improved diagnostic classification for CM, we hope to refine our assessment of CM and its related comorbidities in future studies.

## Conclusion

Despite these limitations, findings of increased RR for CM in the present study were similar to those reported in other studies. The present study confirmed the association between CM and hyperlipidemia, asthma and psychiatric disorders when compared with other migraines, as well as its association with certain metabolic syndrome, respiratory, neurological and psychiatric disorders when compared with the non-migraineurs.

The presence of a comorbid disease may alter the nature, progression and response to treatment of CM and hence should be addressed simultaneously by treating the underlying etiology or implementing preventive measures, where applicable. It is also important for clinicians to recognize CM as a condition that is distinct from other migraines and to formulate a management plan that aims to improve patient outcomes, treatment adherence and quality of life.
